# Non-native *Brachiaria humidicola* with biological nitrification inhibition capacity stimulates *in situ* grassland N_2_O emissions

**DOI:** 10.3389/fmicb.2023.1127179

**Published:** 2023-03-17

**Authors:** Lu Xie, Deyan Liu, Zengming Chen, Yuhui Niu, Lei Meng, Weixin Ding

**Affiliations:** ^1^State Key Laboratory of Soil and Sustainable Agriculture, Institute of Soil Science, Chinese Academy of Sciences, Nanjing, China; ^2^University of Chinese Academy of Sciences, Beijing, China; ^3^College of Tropical Crops, Hainan University, Haikou, China

**Keywords:** biological nitrification inhibition, *Brachiaria humidicola*, denitrification, N_2_O emissions, yield-scaled N_2_O emission

## Abstract

**Introduction:**

*Brachiaria humidicola*, a tropical grass, could release root exudates with biological nitrification inhibition (BNI) capacity and reduce soil nitrous oxide (N_2_O) emissions from grasslands. However, evidence of the reduction effect *in situ* in tropical grasslands in China is lacking.

**Methods:**

To evaluate the potential effects of *B*. *humidicola* on soil N_2_O emissions, a 2-year (2015–2017) field experiment was established in a Latosol and included eight treatments, consisting of two pastures, non-native *B*. *humidicola* and a native grass, *Eremochloa ophiuroide*, with four nitrogen (N) application rates. The annual urea application rates were 0, 150, 300, and 450 kg N ha^−1^.

**Results:**

The average 2-year *E*. *ophiuroides* biomass with and without N fertilization were 9.07–11.45 and 7.34 t ha^−1^, respectively, and corresponding values for *B*. *humidicola* increased to 31.97–39.07 and 29.54 t ha^−1^, respectively. The N-use efficiencies under *E*. *ophiuroide* and *B*. *humidicola* cultivation were 9.3–12.0 and 35.5–39.4%, respectively. Annual N_2_O emissions in the *E*. *ophiuroides* and *B*. *humidicola* fields were 1.37 and 2.83 kg N_2_O-N ha^−1^, respectively, under no N fertilization, and 1.54–3.46 and 4.30–7.19 kg N_2_O-N ha^−1^, respectively, under N fertilization.

**Discussions:**

According to the results, *B*. *humidicola* cultivation increased soil N_2_O emissions, especially under N fertilization. This is because *B*. *humidicola* exhibited the more effective stimulation effect on N_2_O production *via* denitrification primarily due to increased soil organic carbon and exudates than the inhibition effect on N_2_O production *via* autotrophic nitrification. Annual yield-scaled N_2_O emissions in the *B*. *humidicola* treatment were 93.02–183.12 mg N_2_O-N kg^−1^ biomass, which were significantly lower than those in the *E*. *ophiuroides* treatment. Overall, our results suggest that cultivation of the non-native grass, *B*. *humidicola* with BNI capacity, increased soil N_2_O emissions, while decreasing yield-scaled N_2_O emissions, when compared with native grass cultivation.

## 1. Introduction

Nitrous oxide (N_2_O) is a potent greenhouse gas with a significant 100-year global warming potential that is 265 times higher than that of carbon dioxide on a per-molecule basis (IPCC, 2013). In addition, N_2_O depletes stratospheric ozone, which protects the earth from biologically damaging ultraviolet radiation (Ravishankara et al., 2009). Notably, the concentration of atmospheric N_2_O has increased from 270 ppb during the pre-industrial era to 335.55 ppb in 2022, with an average annual increase rate of 0.90 ppb over the last 2 decades (Lan et al., 2022). Agriculture reportedly emitted approximately 4.1 Tg N_2_O-N year^−1^, accounting for approximately 66% of total global anthropogenic N_2_O emissions (UNEP, 2013). Using the dynamic land ecosystem model, Dangal et al. (2019) estimated that the net N_2_O emission from the global grasslands was 2.2 Tg N_2_O-N year^−1^, which was responsible for 54% of the total agricultural N_2_O emissions.

To meet the increasing food demands, nitrogen (N) fertilizer and agricultural land are growing substantially (Foley et al., 2011). The global synthetic N fertilizer consumption has increased from 12 to 112 Tg N while that has risen from 0.8 to 24 Tg N in China during the 1961–2020.^1^ However, the N-use efficiency (NUE) in China was only 28–35%, which is much lower than the global average (Liu et al., 2013; Han et al., 2015; Zhang et al., 2015).The heavy reliance of N fertilizers in agriculture has contributed to the stimulation of nitrifier activity and the trend toward high-nitrifying soil environments (Poudel et al., 2002; Bellamy et al., 2005).

Nitrification is closely related to N utilization and loss, and has become a key process to improve NUE and reduce N pollution (Subbarao et al., 2006; Beeckman et al., 2018). Nitrification is a microbes-driven process of oxidizing ammonia (NH_3_) to nitrite and further to nitrate (NO_3_^−^) and producing N_2_O as a byproduct (Stein, 2020). The NO_3_^−^ produced during nitrification serves as a substrate and denitrification further reduces NO_3_^−^ to dinitrogen and produces N_2_O as an intermediate product (Coskun et al., 2017a). Nitrification inhibitors can depress the activities of nitrifiers in soil, thereby delaying NH_3_ oxidation and reducing N_2_O emissions and NO_3_^−^ production (Rodgers, 1986; Coskun et al., 2017b). To date, a few synthetic nitrification-inhibiting compounds have been efficiently adopted in the field, such as nitrapyrin, dicyandiamide, and 3,4-dimethyl pyrazole phosphate (Weiske et al., 2001; Zerulla et al., 2001; Niu et al., 2018). Meta-analysis revealed that the combination application of nitrification inhibitors and urea reduced NO_3_^−^ leaching by 48% and N_2_O emissions by 44% (Burzaco et al., 2014), and increased crop yields by 7.5% and NUE by 12.9% (Abalos et al., 2014a). However, synthetic nitrification inhibitors have certain limitations such as low cost-effectiveness, application challenges, poor biological stability, and environmental pollution risks (Subbarao et al., 2012; Coskun et al., 2017b; Wang et al., 2021).

Natural compounds with biological nitrification inhibition (BNI) have been found from litters, root exudates, tissue extracts, and rhizosphere of plants such as grasses, trees, and crops (Wang et al., 2021), including methyl 3-(4 hydroxyphenyl) propionate (MHPP), sorgoleone and sakuranetin from sorghum (Subbarao et al., 2013), 1,9-decanediol from rice (Sun et al., 2016) and brachialactone from *Brachiaria humidicola* grass (Subbarao et al., 2009). Some root-secreted biological nitrification inhibitors (BNIs) like sorgoleone, sakuranetin, and brachialactone as well as linolenic acid and linoleic acid found in *B*. *humidicola* can inhibit both ammonia mono-oxygenase and hydroxylamine oxidoreductase activities (Coskun et al., 2017b), while 1,9-decanediol and MHPP only inhibits activity of ammonia mono-oxygenase (Zakir et al., 2008; Nardi et al., 2013, 2020; Sun et al., 2016; Lu et al., 2019). Up to date, the functional validation of the BNIs is mainly performed in the pure culture system of a single strain *Nitrosomonas europaea*, and the effect in the complicated soil system remains to be tested (Subbarao et al., 2015). For example, sakuranetin isolated from sorghum shows a strong inhibitory activity *in vitro*-cultural bioassay but losses the inhibitory effect in soil-assay (Subbarao et al., 2013). Gopalakrishnan et al. (2009) found that the inhibition effect of BNIs is affected by soil type, and the BNIs derived from *B*. *humidicola* in Cambisol can inhibit 90% nitrification with comparable effects to dicyandiamide (50 mg kg^−1^ soil), but are less effective in Andosol during the 60-day incubation.

Forage grasses with biological nitrification inhibition (BNI) capacity exhibit approximately 2-fold greater productivity than those lacking such capacity in nutrient-limited ecosystems, based on an estimate of a newly developed model (Lata et al., 1999; Boudsocq et al., 2009). The *B*. *humidicola*, reportedly exhibits the strongest BNI function among tropical grasses reduces the NH_3_ oxidation rate and N_2_O emissions significantly during a 3-year field experiment, when compared with soybean-planted or plant-free soils (Subbarao et al., 2009). During a short-term (29 days) monitoring period in Colombia, cumulative N_2_O emissions from a *B*. *humidicola* cv. Tully field was decreased by approximately 60% when compared with that in a *Brachiaria* hybrid cv. Mulato field under bovine urine amendment (Byrnes et al., 2017). In contrast, no significant effect on N_2_O emissions of the two forage genotypes was observed under cattle dung amendment in the same experimental site (Lombardi et al., 2022).

Latosol is a most widely distributed soil and covers 51.26% of the total area in Hainan Province, China. In the present study, a 2-year field experiment was conducted in a Latosol cultivated with *B*. *humidicola* and a native grass species, *Eremochloa ophiuroides*. We hypothesized that *in situ* N_2_O emissions from grasslands under cultivation with *Brachiaria* with higher BNI capacity are lower than in those cultivated with *Eremochloa*. The objectives of the present study were: (1) to determine whether the N_2_O emissions from a *B*. *humidicola* field are lower than those from an *E*. *ophiuroides* field in tropical Hainan Province, China; and (2) to evaluate the mitigation effects of *B*. *humidicola* on yield-scaled N_2_O emissions under the different N application rates. We also established an incubation with soils from the field experiment using a ^15^N tracing technique to evaluate the influence of *B*. *humidicola* on the N transformation process rates and N_2_O production rates *via* nitrification and denitrification (Xie et al., 2022).

## 2. Materials and methods

### 2.1. Study site

The field site was located in Danzhou, Hainan Province, China (109°29′ E, 19°30′ N). The region is characterized by a tropical monsoon climate, with a rainy season from May to October, and a dry season from November to April. The mean annual temperature and precipitation are 23.1°C and 1,823 mm, respectively. The soil is derived from granite and classified as a Latosol, with a sandy loam texture. Latosol is a most widely distributed soil in Hainan Province. The properties of surface soil (0–20 cm) prior to the field experiment are shown in Table 1.

**Table 1:** Soil properties before field experiment.

BD (g cm^−3^)	Soil pH	SOC (g C kg^−1^)	TN (g N kg^−1^)	NH_4_^+^-N (mg N kg^−1^)	NO_3_^−^-N (mg N kg^−1^)	Available P (mg P kg^−1^)	Available K (mg K kg^−1^)
1.29 ± 0.18	5.42 ± 0.02	5.70 ± 0.05	0.27 ± 0.01	0.22 ± 0.06	6.03 ± 0.17	20.76 ± 1.06	76.00 ± 3.45

Means ± standard errors (*n* = 3). BD, soil bulk density; SOC, soil organic carbon; TN, total soil nitrogen; Available P, available phosphorus; and Available K, available potassium.

### 2.2. Experimental design

A field experiment was established in August 2015 and included eight treatments, consisting of two pastures, *Brachiaria humidicola* CIAT679 and *Eremochloa ophiuroides*, with four N application rates. The annual urea application rates were 0, 150, 300, and 450 kg N ha^−1^, which were designated as BCK, BN1, BN2, and BN3, respectively, for *B*. *humidicola*, and ECK, EN1, EN2, and EN3, respectively, for *E*. *ophiuroides*. The plots measured 3 m × 4 m. The treatments, which had three replicates, were set up based on a randomized complete block design. During the first season from August 2015 to August 2016, 60 and 40% of urea was applied as basal fertilizer and top-dressing fertilizer, respectively, in the fertilized treatments. In the second season from August 2016 to August 2017, urea was added with three splits: 40% as basal fertilizer, and 30% as top-dressing fertilizer on 13 March and 9 June 2017, respectively. Calcium superphosphate (150 kg P_2_O_5_ ha^−1^) and potassium chloride (105 kg K_2_O ha^−1^) were applied as basal fertilizer. All the fertilizers were dissolved in water and uniformly spread into the soil. Harvested plant samples were oven-dried at 60°C to a constant weight, and then ground to less than 0.2 mm for analysis. Field management practices were similar to local practices and standardized at all plots. Specific dates are listed in Table 2.

**Table 2:** Specific dates of field management during the 2-year field experiment.

Year	Planting	Basal fertilization	Top-dressing fertilization	Harvest
2015–2016	15 August	15 Aug. 2015	15 Apr. 2016	14 April 2016; 27 Aug. 2016
2016–2017	-	28 Aug. 2016	13 Mar. 2017; 9 June 2017	12 Mar. 2017; 8 June 2017; 1 Sept. 2017

### 2.3. Nitrous oxide flux measurement

Nitrous oxide fluxes were measured using the static chamber method. Before grass planting, a stainless steel chamber with a rectangular base (50 cm × 50 cm × 15 cm) and a 5-cm groove around the upper edge was permanently fit 10 cm into the soil. During gas sampling, a stainless chamber (50 cm × 50 cm × 50 cm) was inserted into the groove, which was filled with water to ensure airtightness. The chamber was covered with reflective film and foam to minimize air temperature change inside the chamber. A rubber plug with a mercury thermometer was fit tightly into the hole on the top of the chamber for use in measuring the chamber temperature while gas sampling. Two vents welded with stainless tubes were punched on top of the chamber, one connected to a rubber tube with a three-way stopcock for gas collection, and another one for ensuring air pressure equilibrium inside and outside the chamber. Gas samples were obtained once every other day during 1 week after each fertilization and once a week during the other period. Sampling was conducted between 7:00 am and 12:00 pm to minimize diurnal variation. Four gas samples were extracted from the chamber at 0, 10, 20, and 30 min after chamber closure using airtight plastic syringes and instantly injected into 20-ml pre-evacuated vials fitted with butyl rubber stoppers. The N_2_O concentrations were analyzed using a gas chromatograph (GC; Agilent 7890, Agilent Technologies, Santa Clara, CA, United States) equipped with a ^63^Ni electron capture detector and a thermal conductivity detector. The N_2_O fluxes were calculated using the following equation (Niu et al., 2018):


$$F=\rho \times \left(V/S\right)\times \left(\Delta C/\Delta t\right)\times \left[273/\left(273+T\right)\right]$$

where *F* is the flux in N_2_O (μg N_2_O-N m^−2^ h^−1^); *ρ* is the density of N_2_O at 0°C and 760 mm Hg (kg m^−3^); *V* is the effective volume of the chamber (m^3^); *S* is the soil area covered by the chamber (m^2^); *ΔC*/*Δt* is the rate of N_2_O concentration increase in the chamber (ppbv N_2_O-N h^−1^); and, *T* is mean air temperature inside the chamber during sampling (°C).

### 2.4. Auxiliary variables measurement

Soil temperature was measured at 5 cm depth using a geothermometer. Soil water content was measured at three different positions in each plot with time domain reflectometry (TDR) probes and expressed as water-filled pore space (WFPS, %) as follows (Niu et al., 2018):


WFPS=volumetric water content/total soil porosity

where total soil porosity = 1 - (soil bulk density/2.65), 2.65 being the soil particle density (g cm^−3^).

Surface (0–20 cm) soil samples were collected at five different positions in each plot fortnightly using a stainless steel soil sampler and thoroughly mixed to form a composite sample. The samples were taken to the laboratory immediately and stored at −20°C before analysis. Soil exchangeable ammonium-N (NH_4_^+^-N) and nitrate-N (NO_3_^−^-N) were extracted with 2 M KCl (soil/KCl solution ratio of 1:5) by agitating for 1 h on a rotary shaker, and the concentrations were measured using a colorimetric method on a segmented flow analyzer (Skalar, The Netherlands; Chen et al., 2014). Dissolved organic C (DOC) was extracted with deionized water at a soil water ratio of 1:5, which was shaken for 0.5 h, followed by centrifugation for 15 min at 2,300 × *g* and filtration (<0.45 μm). Subsequently, the DOC was analyzed using the combustion oxidation nondispersive infrared absorption method on a TOC analyzer (vario TOC Cube, Elementar, Hanau, Germany).

Soil samples were collected after the end of the field experiments. Soil pH was determined from soil-water suspensions (1:2.5 v/v) using a pH meter (SevenCompact, Mettler Toledo, Swiss). Soil organic C (SOC) was measured using the wet oxidation-redox titration method (Walkley and Black, 1934). Total N content in soil and plant was determined using an elemental analyzer (Vario MAX, Elementar, Germany). Soil available P was extracted with 0.05 M HCl and 0.025 M H_2_SO_4_, and determined using the molybdenum blue colorimetric method (Ye et al., 2019). Available K was extracted with ammonium acetate and analyzed using a flame photometer (Lu, 2000).

### 2.5. Data calculation and statistical analysis

Annual cumulative N_2_O emissions (*E*_N2O_, kg N_2_O-N ha^−1^) were calculated using the following equation (Chen et al., 2014):


$$E_{N2O}=\overset{n}{\underset{i=0}{\sum }}\left(f_{i}+f_{i+1}\right)/2\times \left(t_{i+1}-t_{i}\right)\times 24\times 10^{-5}$$

where *f* is the N_2_O flux (μg N_2_O-N m^−2^ h^−1^); *i* is the *i*th measurement; (*t*_i + 1_ –*t*_i_) is the interval between the i th and the (*i* + 1)th measurement time (d); *n* is the total number of measurements; and 24 × 10^−5^ was used for unit conversion.

The N_2_O emission factor of applied fertilizer N (EF, %) was calculated using the following equation:


$$EF=\left(E_{fertilizer}-E_{control}\right)/N_{applied}$$

where *E*_fertilizer_ and *E*_control_ are the cumulative N_2_O emissions from the fertilized and control treatments, respectively; and *N*_applied_ is the amount of fertilizer N applied to the corresponding treatment.

The yield-scaled N_2_O emission (mg N_2_O-N kg^−1^ biomass) was computed using the following equation (Venterea et al., 2011):


$$Yield-scaledN_{2}O=E_{N2O}/yield$$

where *E*_N2O_ is the annual cumulative N_2_O emissions (kg N_2_O-N ha^−1^); and yield is the amount of grass biomass harvested annually (kg ha^−1^).

Fertilizer N-use efficiency (NUE, %) was calculated as follows:


$$NUE=\left(N_{fertilizer}-N_{control}\right)/N_{applied}$$

where *N*_fertilizer_ and *N*_control_ are the amount of N uptake in aboveground biomass (kg N ha^−1^) in the fertilized and control treatments, respectively; and *N*_applied_ is the amount of the N applied to the corresponding treatment (kg N ha^−1^).

Significant differences among treatments were evaluated using one-way ANOVA followed by the Duncan test at *p* < 0.05. Spearman’s correlation analysis was used to determine the relationships between N_2_O flux and soil WFPS, soil inorganic N, soil dissolved organic C, and air temperature. All statistical analyses were performed using IBM SPSS Statistics 26 for Windows (IBM corp., Armonk, NY, United States).

## 3. Results

### 3.1. Soil characteristics

After 2 years of grass cultivation, soil pH in all the treatments increased when compared with that in the pre-treatment soil (Table 3). The maximum soil pH was observed in the ECK treatment without N fertilization, and soil pH decreased with an increase in N application rate in both grasslands. SOC increased by 17.5–22.8% under *B*. *humidicola* cultivation and only by 5.8–15.1% under *E*. *ophiuroides*, when compared with the pre-treatment soil. Cultivation of both pastures promoted soil N accumulation significantly (*p* < 0.05); however, there were no significant differences in soil N accumulation among treatments under different N application rates (Table 3).

**Table 3:** Soil properties before and after 2 years of *Brachiaria humidicola* and *Eremochloa ophiuroides* cultivation.

Treatment	Soil pH	SOC (g C kg^−1^)	TN (g N kg^−1^)	DOC (mg C kg^−1^)
Pre-soil	5.42 ± 0.02d	5.70 ± 0.05b	0.27 ± 0.01b	115.87 ± 7.39d
BCK	6.37 ± 0.17a	6.70 ± 0.19ab	0.52 ± 0.03a	161.45 ± 3.70b
BN1	6.41 ± 0.27a	6.78 ± 0.65ab	0.55 ± 0.02a	151.48 ± 1.95bc
BN2	5.75 ± 0.05bcd	6.89 ± 0.10a	0.55 ± 0.02a	142.71 ± 8.68c
BN3	5.60 ± 0.07 cd	7.00 ± 0.30a	0.58 ± 0.04a	145.40 ± 0.66c
ECK	6.49 ± 0.16a	6.03 ± 0.00ab	0.51 ± 0.01a	160.15 ± 3.53bc
EN1	6.06 ± 0.10abc	6.51 ± 0.48ab	0.59 ± 0.05a	147.11 ± 8.22c
EN2	6.19 ± 0.15ab	6.56 ± 0.08ab	0.57 ± 0.04a	156.53 ± 1.79bc
EN3	5.74 ± 0.16bcd	6.48 ± 0.38ab	0.55 ± 0.04a	179.84 ± 6.56a

Means ± standard errors (*n* = 3). BCK, no nitrogen application for *B*. *humidicola*; BN1, nitrogen application at 150 kg N ha^−1^ for *B*. *humidicola*; BN2, nitrogen application at 300 kg N ha^−1^ for *B*. *humidicola*; BN3, nitrogen application at 450 kg N ha^−1^ for *B*. *humidicola*; ECK, no nitrogen application for *E*. *ophiuroides*; BN1, nitrogen application at 150 kg N ha^−1^ for *E*. *ophiuroides*; EN2, nitrogen application at 300 kg N ha^−1^ for *E*. *ophiuroides*; EN3, nitrogen application at 450 kg N ha^−1^ for *E*. *ophiuroides*. Pre-soil, soil prior to field experiment; SOC, soil organic carbon; TN, total soil nitrogen; DOC, dissolved organic carbon. Different letters within the same columns indicate significant differences between treatments (*p* < 0.05).

### 3.2. Grass yield

The biomass of *B*. *humidicola* ranged from 29.54 to 31.37 t ha^−1^ in the BCK treatment, which was 3.1–6.0-fold that of *E*. *ophiuroide* during the two seasons (Table 4). The N application increased biomass yield of *B*. *humidicola* by 11.3–25.8% (*p* < 0.05) but did not increase the biomass yield of *E*. *ophiuroides*, during the first season. During the 2016–2017 season, however, the biomass of both grasses was enhanced with N fertilization, and increased with an increase in the N application rate (*p* < 0.05).

**Table 4:** Yield, nitrogen uptake, and nitrogen use efficiency of *Brachiaria humidicola* and *Eremochlo*a *ophiuroides* during two growth seasons.

Treatment	2015–2016			2016–2017			Mean		
	Yield (t ha^−1^)	N uptake (kg N ha^−1^)	NUE (%)	Yield (t ha^−1^)	N uptake (kg N ha^−1^)	NUE (%)	Yield (t ha^−1^)	N uptake (kg N ha^−1^)	NUE (%)
BCK	31.37 ± 0.30c	220.08 ± 8.57b	–	29.54 ± 0.14d	188.74 ± 17.65d	–	30.45 ± 0.21c	204.41 ± 11.96d	–
BN1	34.91 ± 2.05b	249.37 ± 10.77b	19.5 ± 6.1ab	31.92 ± 0.62c	277.47 ± 9.81c	59.2 ± 5.3a	33.41 ± 0.81b	263.42 ± 5.76c	39.3 ± 5.5a
BN2	38.72 ± 0.73a	308.59 ± 23.54a	29.5 ± 10.6a	37.01 ± 0.76b	336.02 ± 5.84b	49.1 ± 6.5a	37.87 ± 0.73a	322.3 ± 13.79b	39.3 ± 8.4a
BN3	39.47 ± 0.60a	324.02 ± 12.00a	23.1 ± 0.8ab	39.07 ± 0.46a	404.07 ± 7.22a	47.9 ± 3.5a	39.27 ± 0.43a	364.05 ± 5.74a	35.5 ± 1.4a
ECK	5.24 ± 0.51d	44.22 ± 2.25c	–	9.44 ± 0.15 g	69.82 ± 1.63 g	–	7.34 ± 0.31f	57.02 ± 1.12f	–
EN1	7.47 ± 0.34d	59.90 ± 3.33c	10.5 ± 3.0b	10.66 ± 0.82 g	90.08 ± 1.59 fg	13.5 ± 0.3b	9.07 ± 0.58e	74.99 ± 2.45f	12.0 ± 1.4b
EN2	6.50 ± 0.19d	63.11 ± 4.27c	6.3 ± 1.5b	12.77 ± 0.44f	106.68 ± 2.88f	12.3 ± 0.7b	9.63 ± 0.31e	84.9 ± 1.18ef	9.3 ± 0.5b
EN3	7.74 ± 0.61d	72.15 ± 2.99c	6.2 ± 1.1b	15.15 ± 0.18e	138.07 ± 7.95e	15.2 ± 2.1b	11.45 ± 0.22d	105.11 ± 2.48e	10.7 ± 0.6b

Means ± standard errors (*n* = 3). BCK, no nitrogen application for *B*. *humidicola*; BN1, nitrogen application at 150 kg N ha^−1^ for *B*. *humidicola*; BN2, nitrogen application at 300 kg N ha^−1^ for *B*. *humidicola*; BN3, nitrogen application at 450 kg N ha^−1^ for *B*. *humidicola*; ECK, no nitrogen application for *E*. *ophiuroides*; BN1, nitrogen application at 150 kg N ha^−1^ for *E*. *ophiuroides*; EN2, nitrogen application at 300 kg N ha^−1^ for *E*. *ophiuroides*; EN3, nitrogen application at 450 kg N ha^−1^ for *E*. *ophiuroides*. Yield, grass aboveground biomass; N uptake, the amount of N uptake in aboveground biomass; NUE, nitrogen use efficiency. Different letters in the same column indicate significant differences between treatments (*p* < 0.05).

The amounts of N uptake by *B*. *humidicola* under no N fertilization were 220.08 and 188.74 kg N ha^−1^ during the 2015–2016 and 2016–2017 seasons, respectively, and decreased to 44.22 and 69.82 kg N ha^−1^ for *E*. *ophiuroide*, respectively (Table 4). The mean NUE of the N applied under *B*. *humidicola* was 19.5–29.5% during the 2015–2016 season and increased to 47.9–59.2% during the 2016–2017 season, which was significantly higher than that under *E*. *ophiuroides* during both seasons.

### 3.3. Soil and environmental variables

**Figure 1:**
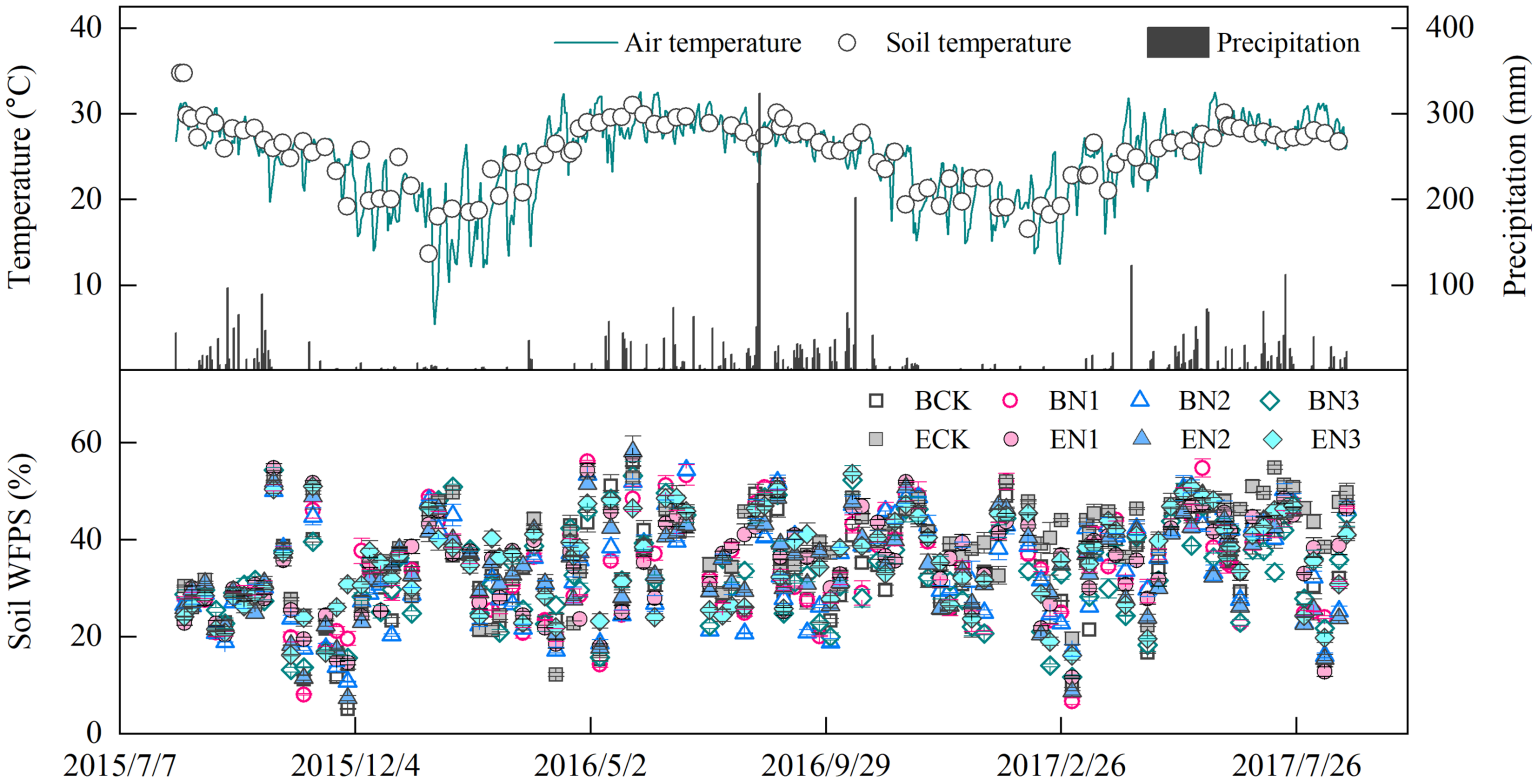
Temporal variation in precipitation, air temperature, and soil temperature at 5 cm depth, and water-filled pore space (WFPS). Vertical bars denote the standard errors of the mean (*n* = 3).

Air temperature (AT) ranged from 5.4 to 32.6°C, with an average of 24.8°C over the 2-year measurement period, and there was no apparent difference between two growth seasons (Figure 1). Soil temperature (ST) at 5 cm depth ranged from 13.7 to 34.8°C, a trend similar to that of AT (ST = 0.758AT + 7.062, *R*^2^ = 0.42, *p* < 0.01). Mean rainfall was 2,341 and 2,373 mm during the 2015–2016 and 2016–2017 seasons, respectively. Precipitation mainly occurred in the rainy season, from May to October, accounting for 87% of the total annual precipitation. Soil moisture fluctuated from 5.1 to 58.3% WFPS, and the mean WFPS in all the treatments was 33.5–38.3%, with no significant differences among treatments.

**Figure 2:**
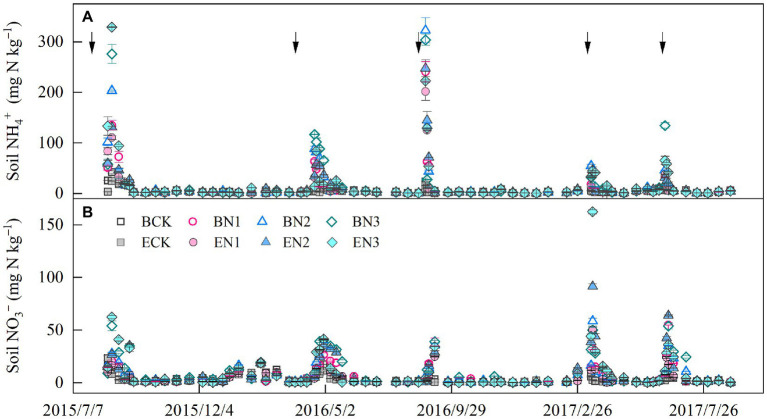
Soil ammonium **(A)** and nitrate **(B)** concentration dynamics in the 0–20-cm layer. Vertical bars denote the standard errors of the mean (*n* = 3). The solid arrows indicate the N fertilization time.

Soil NH_4_^+^-N concentration peaks occurred approximately 1 week after each fertilization, and decreased to a constant level 40 days later (Figure 2A). The mean soil NH_4_^+^-N concentrations under the BCK and ECK treatments were 4.60 and 4.05 mg N kg^−1^, respectively and increased to 10.46–14.93 and 10.09–15.91 mg N kg^−1^ in the BN and EN treatments, respectively, increasing with increases in the N application rate. Mean soil NH_4_^+^-N concentrations were not significantly different between the *B*. *humidicola* and *E*. *ophiuroides* fields under similar N application rates. Soil NO_3_^−^-N concentrations in the BCK and ECK treatments were on average 3.21 and 2.59 mg N kg^−1^, respectively (Figure 2B). Under N fertilization, mean soil NO_3_^−^-N concentrations increased to 5.61, 6.02, and 8.42 mg N kg^−1^ in the BN1, BN2, and BN3 treatments, respectively, which were higher than the corresponding values under *E*. *ophiuroides* cultivation, excluding BN2 (*p* < 0.05).

### 3.4. Nitrous oxide emissions

**Figure 3:**
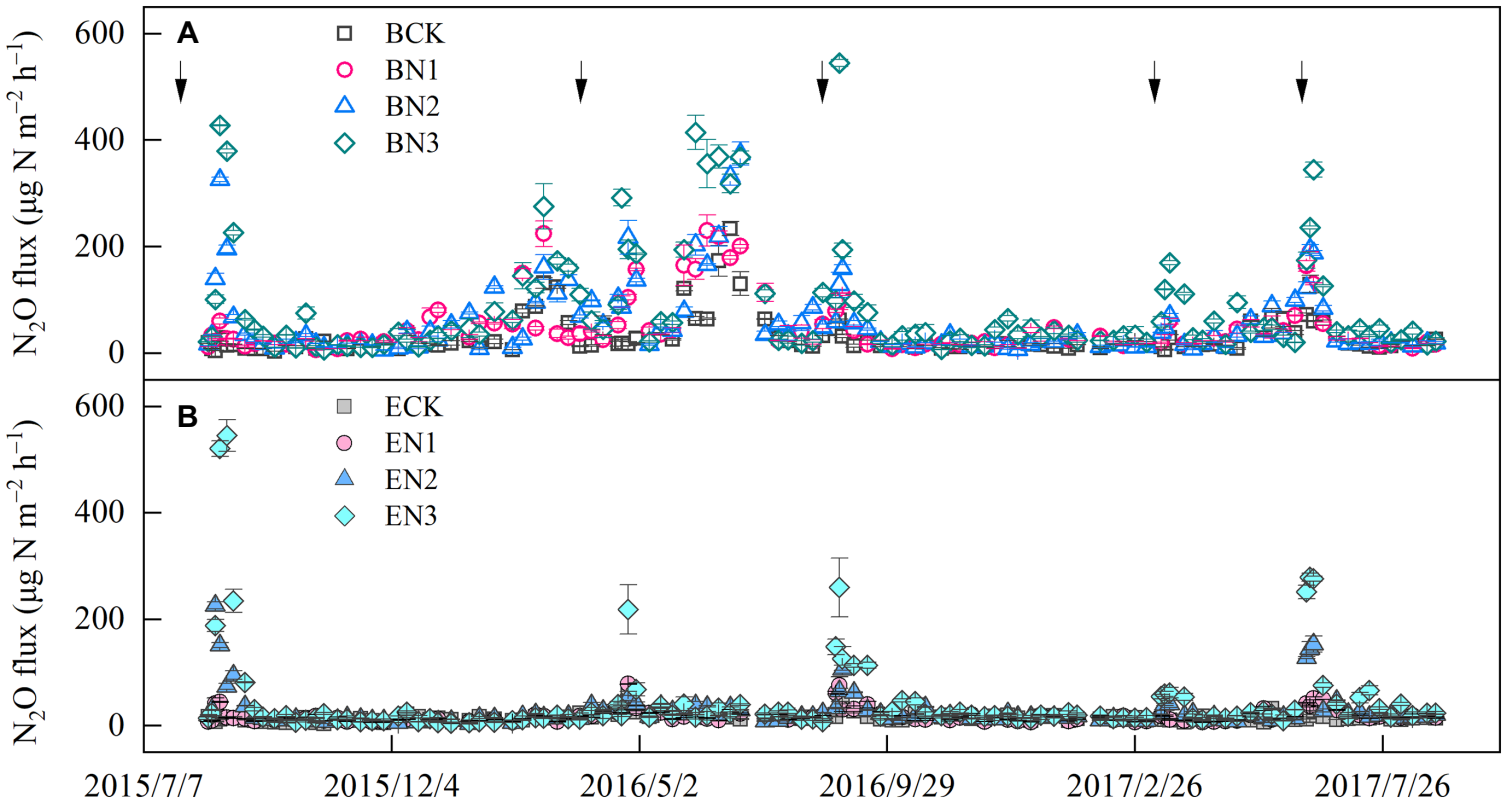
Temporal variation in nitrous oxide (N_2_O) flux in *Brachiaria humidicola* **(A)** and *Eremochloa ophiuroides* **(B)** soil. Solid line arrows indicate the timing of fertilizer application. Vertical bars denote the standard errors of the mean (*n* = 3). The solid arrows indicate the nitrogen (N) fertilization time.

Nitrous oxide flux peaks emerged after each fertilization, and increased with an increase in the N application rates. The highest flux was 544.60 μg N_2_O-N m^−2^ h^−1^ in the BN3 treatment on 31 August 2016, which was 3-fold that in the EN3 treatment (Figure 3). During the 2016–2017 season, the peak flux in the BN3 treatments (344.60 μg N_2_O-N m^−2^ h^−1^) was also observed on 20 June 2017, which, however, was only 1.2-fold greater than that in the EN3 treatment. The N_2_O fluxes were significantly (*p* < 0.01) correlated with soil moisture, NH_4_^+^-N, NO_3_^−^-N, and air temperature (Table 5).

**Table 5:** Correlation between nitrous oxide (N_2_O) flux and soil moisture (WFPS), ammonium-nitrogen (NH_4_^+^-N), nitrate-nitrogen (NO_3_^−^-N), inorganic nitrogen (NH_4_^+^-N plus NO_3_^−^-N), and air temperature (AT).

Treatment	WFPS	NH_4_^+^-N	NO_3_^−^-N	Inorganic N	AT
BCK	0.232^*^	0.348^**^	0.166	0.339^**^	0.289^**^
BN1	0.224^*^	0.325^**^	0.310^**^	0.390^**^	0.224^*^
BN2	0.193^*^	0.335^**^	0.264^**^	0.363^**^	0.415^**^
BN3	0.245^**^	0.290^**^	0.360^**^	0.370^**^	0.238^*^
ECK	0.148	0.113	−0.043	0.030	0.188^*^
EN1	0.171	0.290^**^	0.131	0.248^**^	0.445^**^
EN2	0.208^*^	0.411^**^	0.259^**^	0.358^**^	0.504^**^
EN3	0.200^*^	0.457^**^	0.246^**^	0.379^**^	0.475^**^

^*^p < 0.05, ^**^p < 0.01. BCK, no nitrogen application for *B*. *humidicola*; BN1, nitrogen application at 150 kg N ha^−1^ for *B*. *humidicola*; BN2, nitrogen application at 300 kg N ha^−1^ for *B*. *humidicola*; BN3, nitrogen application at 450 kg N ha^−1^ for *B*. *humidicola*; ECK, no nitrogen application for *E*. *ophiuroides*; BN1, nitrogen application at 150 kg N ha^−1^ for *E*. *ophiuroides*; EN2, nitrogen application at 300 kg N ha^−1^ for *E*. *ophiuroides*; and EN3, nitrogen application at 450 kg N ha^−1^ for *E*. *ophiuroides*.

**Figure 4:**
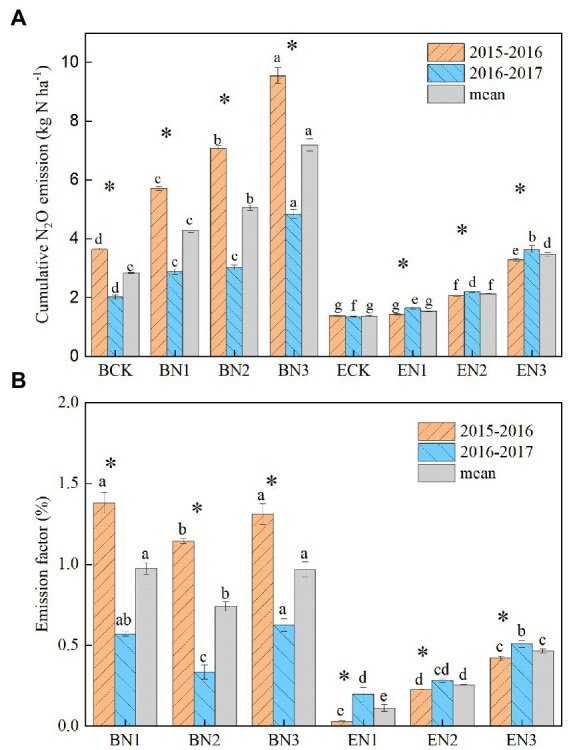
Annual soil nitrous oxide (N_2_O) emission **(A)** and emission factor of the fertilizer nitrogen applied **(B)** in the *Brachiaria humidicola* and *Eremochloa ophiuroides* fields. Vertical bars denote the standard errors of the mean (*n* = 3). Different letters indicate significant differences between treatments for the same measurement year and mean at *p* < 0.05. ^*^indicates the significant difference between 2 years for the same treatment at *p* < 0.05.

Annual N_2_O emissions in the *B*. *humidicola* fields were higher than those in the *E*. *ophiuroides* fields, regardless of N fertilization rate (*p* < 0.05; Figure 4A). They were also greater during the first (2015–2016) season than during the second (2016–2017) season in the case of *B*. *humidicola* but not in the case of *E*. *ophiuroides*. Annual N_2_O emissions in the *B*. *humidicola* fields under BCK were 3.64 and 2.02 kg N_2_O-N ha^−1^ during the 2015–2016 and 2016–2017 season, respectively, with an average of 2.83 kg N_2_O-N ha^−1^. Under N fertilization, annual N_2_O emissions from the *B*. *humidicola* field increased to 5.72–9.54 and 2.88–4.84 kg N_2_O-N ha^−1^ during the 2015–2016 and 2016–2017 season, respectively. In the *E*. *ophiuroides* field, N_2_O emissions under no N fertilization (ECK) were 1.38 and 1.35 kg N_2_O-N ha^−1^ during the 2015–2016 and 2016–2017 seasons, respectively, and increased to 1.43–3.28 and 1.65–3.64 kg N_2_O-N ha^−1^ under N fertilization, respectively. The annual N_2_O emissions increased linearly with an increase in the N application rate in the *B*. *humidicola* fields (E_N2O_ = 0.0092 N_applied_ + 2.76, *R*^2^ = 0.97); conversely, they exhibited exponential correlations with the N application rate in the *E*. *ophiuroides* fields (E_N2O_ = 1.24e^0.021Napplied^, *R*^2^ = 0.95).

The N_2_O emission factor (EF) of the N applied ranged from 0.74 to 0.98% for *B*. *humidicola*, and decreased to 0.11–0.47% for *E*. *ophiuroides* over the 2 years (Figure 4B). The EF increased with an increase in the N application rate only under *E*. *ophiuroides* cultivation.

### 3.5. Yield-scaled nitrous oxide emissions

**Figure 5:**
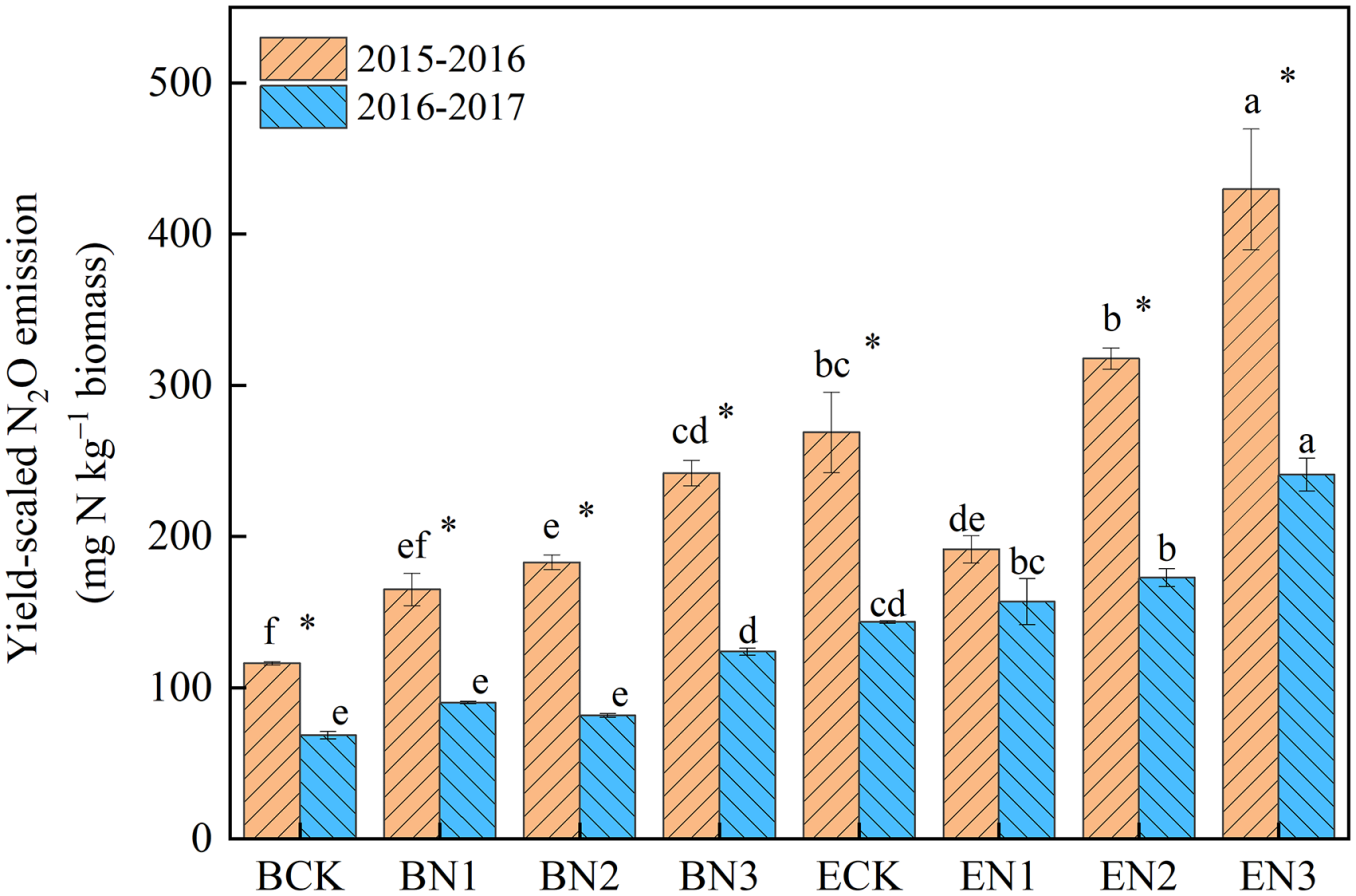
Yield-scaled N_2_O emissions from the *Brachiaria humidicola* and *Eremochloa ophiuroides* field. Vertical bars denote the standard errors of the mean (*n* = 3). Different letters indicate the significant differences between treatments for the same measurement year and for mean at *p* < 0.05. ^*^indicates the significant difference between two measurement years for the same treatment at *p* < 0.05.

The mean yield-scaled N_2_O emissions in the BCK and ECK treatments were 95 and 206 mg N_2_O-N kg^−1^ biomass, respectively, over the 2 years (Figure 5). Under N fertilization, they increased to 128, 132, and 183 mg N_2_O-N kg^−1^ biomass in the BN1, BN2, and BN3 treatments, respectively, which were significantly lower than the corresponding values in the EN treatments by 26.76–46.04%. The reduction increased with an increase in the N application rate.

## 4. Discussion

Annual N_2_O emissions from this tropical grassland varied from 1.35 to 9.54 kg N_2_O-N ha^−1^, which was within the 0–29.1 kg N_2_O-N ha^−1^ range in grasslands as reported previously (Mosier et al., 1996; Wolf et al., 2010; Merbold et al., 2014; Luo et al., 2017). Out of expectation, N_2_O emissions from the *B*. *humidicola* field were 1.3–2.6-fold higher than those from the *E*. *ophiuroides* field under N fertilization. Additionally, the N_2_O emission factor of the N applied was increased to 0.74–0.98% under *B*. *humidicola* from 0.11–0.47% under *E*. *ophiuroides*. Our results suggest that cultivation of exotic, tropical forage grass *B*. *humidicola* with BNI capacity by replacing native *E*. *ophiuroides* stimulated N_2_O emission. To our knowledge, this is the first study to find the stimulation effect of *B*. *humidicola* on N_2_O emissions in the field when compared with the native grass. Apparently, more field studies are required to evaluate the impact of plants with BNI capacity on N_2_O emissions at the ecosystem and global scale, as suggested by Lata et al. (2022).

Previous study suggested that *Brachiaria* genotype with high BNI capacity reduced almost 50% of N_2_O emission when compared with soybean or plant-free soils (Subbarao et al., 2009). Byrnes et al. (2017) reported that *B*. *humidicola* cv. Tully with high BNI capacity reduced approximately 60% of N_2_O emissions in the field when compared with the *Brachiaria* hybrid cv. Mulato having low BNI capacity during the 29-day monitoring period. Planting *B*. *humidicola* with high BNI capacity reduced soil N_2_O emissions by 18.3% when compared with *B*. *humidicola* with low BNI capacity in a 21-day pot experiment (Teutscherová et al., 2022). The active substances with BNI capacity, such as methyl-p-coumarate, methyl ferulate, and brachialactone, have been identified from exudates of *B*. *humidicola* (Gopalakrishnan et al., 2009; Subbarao et al., 2009). Brachialactone can simultaneously block the enzymatic pathways of ammonia monooxygenase and hydroxylamino oxidoreductase (Subbarao et al., 2009). The inhibitory potential reportedly increases with an increase in grass root density (Subbarao et al., 2007; Boudsocq et al., 2009). Subbarao et al. (2009) estimated that *B*. *humidicola* roots can potentially release 2.6 × 10^6^–7.5 × 10^6^ ATU (allylthiourea units) ha^−1^ day^−1^ BNI activity in the South American savannas, which is equivalent to the application of 6.2–18 kg ha^−1^ nitrapyrin based on 1 ATU being equal to 0.6 μg of nitrapyrin. Karwat et al. (2017) demonstrated that *B*. *humidicola,* like dicyandiamide, significantly suppresses soil nitrification potential. In a previous study, using the ^15^N tracing incubation with soils collected from the *B*. *humidicola* and *E*. *ophiuroides* plots at the field experiment end, we found that *B*. *humidicola* decreased the autotrophic nitrification rate and N_2_O production rate *via* nitrification by 27.3 and 14.7%, respectively when compared with *E*. *ophiuroides* (Xie et al., 2022). This indicated that in the test soil, *B*. *humidicola* efficiently inhibited nitrification and N_2_O production *via* nitrification.

Subbarao et al. (2009) observed that cultivation of *B*. *humidicola* reduced the abundance of both ammonia-oxidizing archaea (AOA) and bacteria (AOB) in a Vertisol with pH 7.40 when compared with soil cultivated with soybean. Hink et al. (2018) further reported that although both AOA and AOB were capable of N_2_O production under high NH_4_^+^-N concentrations, the contribution of AOB was greater in a soil with pH 6.50. In the test acid soil with pH 5.42, it is likely that both AOA and AOB participated in NH_3_ oxidation and N_2_O production. Further investigations are required to determine the relative importance of AOA and AOB in N_2_O production, and the suppression effects of *B*. *humidicola* on AOA and AOB activity (Nuñez et al., 2018).

Byrnes et al. (2017) suggested that by increasing N uptake, *B*. *humidicola* with high BNI capacity more efficiently decreased soil NO_3_^−^-N availability and potential denitrification than *B*. *humidicola* with low BNI capacity, thereby reducing N_2_O emissions. Abalos et al. (2014b) reported that mixed cultivation of *Folium perennial* L. and *Poi trivialize* L. decreased soil NO_3_^−^-N concentrations and consequent N_2_O emissions when compared with either monoculture at an N application rate of 60 kg N ha^−1^. They suggested that the trends were attributed to *L*. *perennial* taking up N using the “scale strategy” by increasing root biomass, and *P*. *trivialize* absorbing N *via* the “precision strategy” by providing access to N hotspots that were not emptied by *L*. *perennial*. In the present study, although *B*. *humid cola* cultivation increased N uptake, N_2_O emissions were positively correlated with pasture yield and N uptake, indicating that increased N uptake by *B*. *humid cola* did not reduce N_2_O emissions. In the present study, the mean soil NO_3_^−^-N concentration under N fertilization ranged from 5.60 mg N kg^−1^ in the BN1 treatment to 8.45 mg N kg^−1^ in the BN3 treatment, which was higher than the 5 mg N kg^−1^ threshold for occurrence of denitrification (Dobbie and Smith, 2003), and indicated that although *B*. *humid cola* efficiently increased N uptake and partially inhibited nitrification, soil NO_3_^−^-N under N fertilization was still higher than the threshold value for denitrification in the test field.

Using ^15^N paired incubation (^15^NH_4_NO_3_ and NH_4_^15^NO_3_), we found that the N_2_O production rate during denitrification in the *B*. *humid cola* soil increased by 7.7-fold when compared with the *E*. *ophiuroides* soil and the contribution of denitrification to N_2_O emissions sharply enhanced from 9.7% in the *E*. *ophiuroides* soil to 47.1% (Xie et al., 2022). In the present study, *B*. *humidicola* biomass was 3–6-fold greater than that of *E*. *ophiuroides* and SOC was more efficiently increased under *B*. *humidicola*. Horrocks et al. (2019) also observed that 1-year cultivation of *B*. *humidicola* increases SOC content and improves aggregate stability in Colombia. Fisher et al. (1994) and Amézquita et al. (2004) attributed the SOC enhancement to rapid accumulation of *B*. *humidicola* roots and exudates. Plant reportedly release as much as 40% of net photosynthetic C into the rhizosphere (Marschner, 2011), which in turn provides more labile C substrates for denitrifiers (Wu et al., 2017). Enhanced SOC at least exhibits two stimulation effects on denitrification. Firstly, enhanced SOC promotes the formation of anaerobic microsites for denitrification by stimulating aggregation (Bollmann and Conrad, 2004). Secondly, increased organic C availability reduces the soil moisture threshold for the occurrence of denitrification (Rochette et al., 2000; Van Groenigen et al., 2004; Chantigny et al., 2013) resulting in increased denitrification potentials. Our results indicate that cultivation of exotic *B*. *humidicola* with a much higher biomass compared with *E*. *ophiuroides* stimulated N_2_O production during denitrification by providing more organic C, which in turn masked N_2_O reduction by inhibiting nitrification, thereby enhancing N_2_O emissions.

Comparing yield-scaled N_2_O emissions has been suggested to be an effective way of evaluating the tradeoff between production and environmental impacts and determining the economic feasibility of N_2_O emission mitigation methods (van Groenigen et al., 2010; Grassini and Cassman, 2012). In the present study, yield-scaled N_2_O emissions from *B*. *humidicola* field with and without N fertilization during two seasons were 128.80–183.02 and 93.02 g N kg^−1^ biomass, respectively, which were significantly lower than the corresponding values under *E*. *ophiuroides* cultivation (171.07–221.62 and 186.93 g N kg^−1^ biomass, respectively). In addition, we observed interannual shifts in yield-scaled N_2_O emissions in both grasslands, which was primarily driven by changes in annual N_2_O emission in *B*. *humidicola* fields, whereas they were driven by changes in biomass yield in *E*. *ophiuroides* fields. The lower yield-scaled N_2_O emissions under *B*. *humidicola* cultivation compared with under *E*. *ophiuroides* indicated that although *B*. *humidicola* increased annual N_2_O emissions, it was more environmentally friendly based on its higher forage productivity and NUE.

## 5. Conclusion

**Figure 6:**
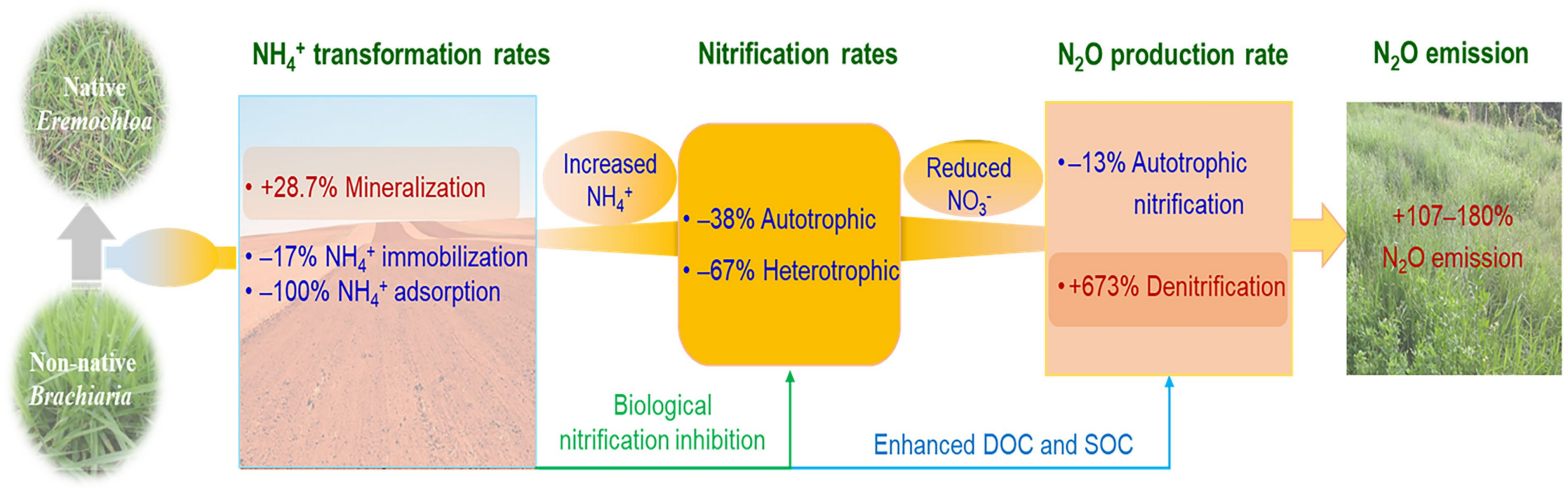
Schematic diagram showing how *Brachiaria humidicola* cultivation stimulated the nitrous oxide (N_2_O) emission in the study grassland. Nitrogen transformation rates and N_2_O production rates *via* autotrophic nitrification and denitrification are cited from Xie et al. (2022).

In the present study, *B*. *humidicola* exhibited higher yields and NUE, and in contrast and unexpectedly, induced higher soil N_2_O emissions when compared with *E*. *ophiuroides*. Although cultivation of *B*. *humidicola* with high BNI capacity reduced N_2_O production rate *via* nitrification, however, it more efficiently enhanced N_2_O production rate than *E*. *ophiuroides via* denitrification due to increased SOC and exudate concentrations, thereby increasing N_2_O emissions (Figure 6). When compared with under *E*. *ophiuroides*, however, the lower yield-scaled N_2_O emissions under *B*. *humidicola* cultivation indicated that although *B*. *humidicola* increased annual N_2_O emissions, it was more environmentally friendly based on its higher forage productivity and NUE.

## Data availability statement

The original contributions presented in the study are included in the article/supplementary material, further inquiries can be directed to the corresponding author.

## Author contributions

WD and DL: conceptualization. LX, YN, and DL: field experiment. LX and ZC: data analysis. LX, WD, and LM: writing. WD: funding acquisition. All authors contributed to the article and approved the submitted version.

## Funding

This work was financially supported from the National Natural Science Foundation of China (41730753, 41977049 and 42077029), the International Partnership Program of Chinese Academy of Sciences (151432KYSB20200001), and International Atomic Energy Agency coordinated research project (D15020).

## Conflict of interest

The authors declare that the research was conducted in the absence of any commercial or financial relationships that could be construed as a potential conflict of interest.

## Publisher’s note

All claims expressed in this article are solely those of the authors and do not necessarily represent those of their affiliated organizations, or those of the publisher, the editors and the reviewers. Any product that may be evaluated in this article, or claim that may be made by its manufacturer, is not guaranteed or endorsed by the publisher.

## Footnotes

^1^https://www.ifastat.org/databases/graph/1
